# The Vicissitudes of Dental Journalism

**Published:** 1902-11

**Authors:** 


### Editorial.

### THE VICISSITUDES OF DENTAL JOURNALISM.

Early in the preceding century the awakening spirit of fraternity and the desire of imparting knowledge of dental art and science led, in 1839, to the issue of the *American Journal of Dental Science.*

This became, considering the small degree of advancement of dental surgery at that time, a remarkable magazine, and it did a great work in moulding the sentiment and cultivating those then in practice. Had its course not been interfered with the probabilities are that it would have maintained its original character. It would have advanced with the development of dental science, and necessarily should have received the required support.

"In 1843 and 1847 the two dental depots then in existence here, conceiving the benefit it would be to them, assumed the liberty each to publish a dental periodical, by which they could secure a hold on the rising profession, thus combining the dissemination of literature contributed to them, and promotion of trade. The evident result of this movement, if not the intention, was the ultimate supplanting of the *American Journal* by offering the new journals at a much lower price."

At length other traders in dental goods came into the field, and, being interdicted from advertising in the trade journals, were obliged to publish their own. Their terms were the same as the others.

The next move affecting the interests of dental literature was the increase in the number of dental depots, brought about by the great profit upon dental goods, each depot deeming it necessary to possess its own journal. These newer journals were put forth at one dollar per year, they virtually being respectable-looking trade-catchers. From the indications, these one dollar people have seriously impaired the interests of the higher-priced journals, and their houses as well. This condition has forced the latter to drop to the lower price. Thus the war among these competitors takes on a new phase. The logical result will be that to outdo each other none of them will be particular to collect their small price; thus dental journalism will be still more dragged down.

It becomes more evident that these journals are simply sugar-coated advertising sheets, which the manufacturers virtually give away rather than not have them circulated.

Ash & Sons' course has been more logical and honorable, in that they freely distribute their quarterly circular. This method will probably become the next step, in case the little dragons continue to eat into the vitals of the larger ones.

The fact becoming more clearly apparent as to the motive governing depot-journalism, the mask being thrown aside, it behooves all who have regard for professional respect and honor to cultivate appreciation of whatever effort may be made towards independent journalism. The dental profession in this country has largely lost its solidarity, and can regain its lost *esprit de corps* by cultivating the liberal principles which actuated the earlier makers of dentistry.

What is most needed is the moral courage to stand shoulder to shoulder in the defence of whatever is right and square in the present and for the promotion of that which is best for the growth of professional honor.

